# Concurrent Rotator Cuff Tear and Axillary Nerve Palsy Associated with Anterior Dislocation of the Shoulder and Large Glenoid Rim Fracture: A “Terrible Tetrad”

**DOI:** 10.1155/2014/312968

**Published:** 2014-06-12

**Authors:** Fumiaki Takase, Atsuyuki Inui, Yutaka Mifune, Tomoyuki Muto, Yoshifumi Harada, Takeshi Kokubu, Masahiro Kurosaka

**Affiliations:** Department of Orthopaedic Surgery, Kobe University Graduate School of Medicine, Kobe 650-0017, Japan

## Abstract

We present a case of concurrent rotator cuff tear and axillary nerve palsy resulting from anterior dislocation of the shoulder and a large glenoid rim fracture—a “terrible tetrad.” A 61-year-old woman fell on her right shoulder. Radiographs showed anterior dislocation of the shoulder with a glenoid rim fracture, and an MRI two months after injury revealed a rotator cuff tear. Upon referral to our hospital, physical and electrophysiological examinations revealed axillary nerve palsy. The axillary nerve palsy was incomplete and recovering, and displacement of the glenoid rim fracture was minimal and already united; therefore, we surgically repaired only the rotator cuff tear three months after injury. The patient recovered satisfactorily following the operation. In patients whose axillary nerve palsy is recovering, surgeons should consider operating on rotator cuff tears in an attempt to prevent rotator cuff degeneration.

## 1. Introduction

Gonzalez and Lopez first reported the combination of peripheral nerve injury and rotator cuff tear following anterior dislocation of the shoulder in 1991 [1]. The combination of pathologies was termed the “unhappy triad” of the shoulder in 1994 [2], and the “terrible triad” of the shoulder in 1995 [3]. The incidence of the “terrible triad” was assumed to be 9–18% of all shoulder dislocations [4]. There has been no reported case of the “terrible triad" with concomitant glenoid rim fracture. We report a unique case of concurrent rotator cuff tear and axillary nerve palsy associated with anterior dislocation of the shoulder and a large glenoid rim fracture—“a terrible tetrad” as we defined.

## 2. Case Presentation

**Figure 1:**
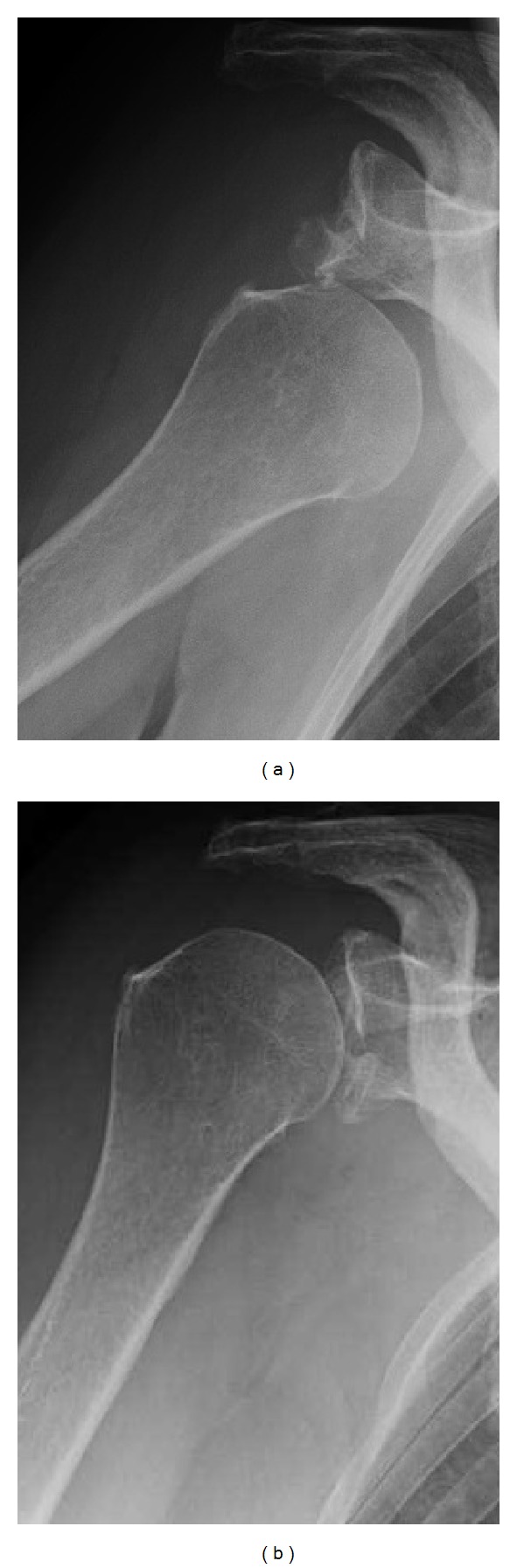
(a) X-ray imaging at the time of injury. (b) X-ray imaging after reduction of anterior shoulder dislocation.

**Figure 2:**
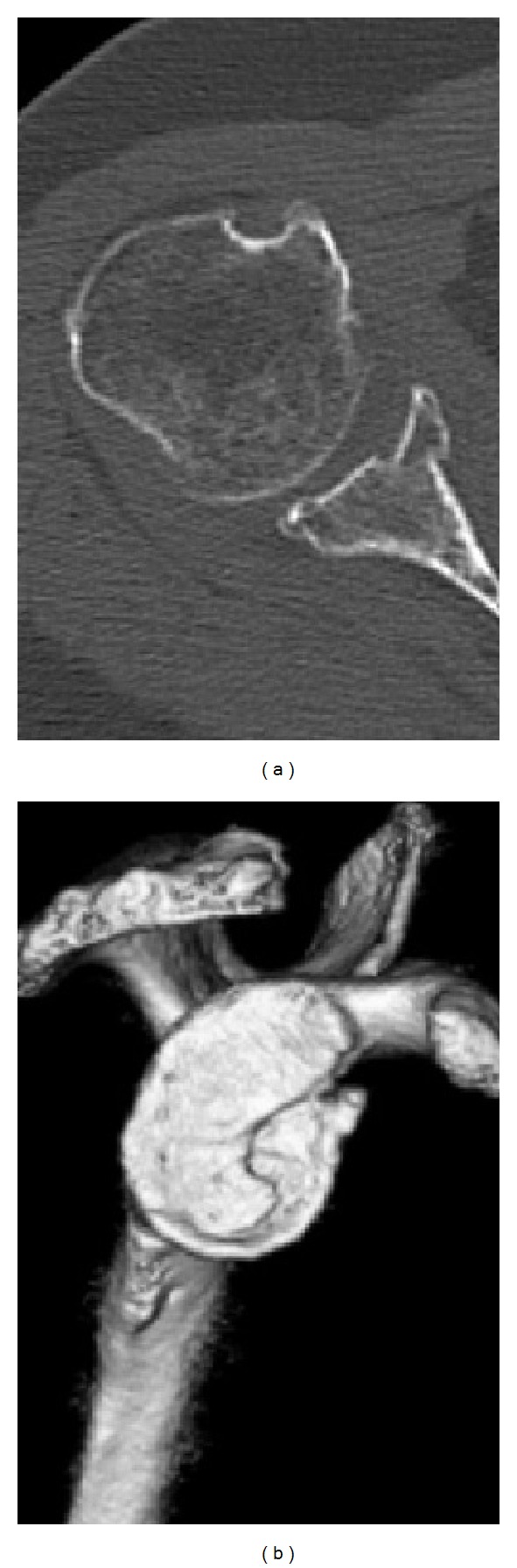
CT imaging upon initial presentation at our hospital. (a) Axial CT imaging showing a large glenoid rim fracture and an articular step-off of 2.5 mm. (b) The width of the bony fragment was 30% of the glenoid length on the three-dimensional CT.

**Figure 3:**
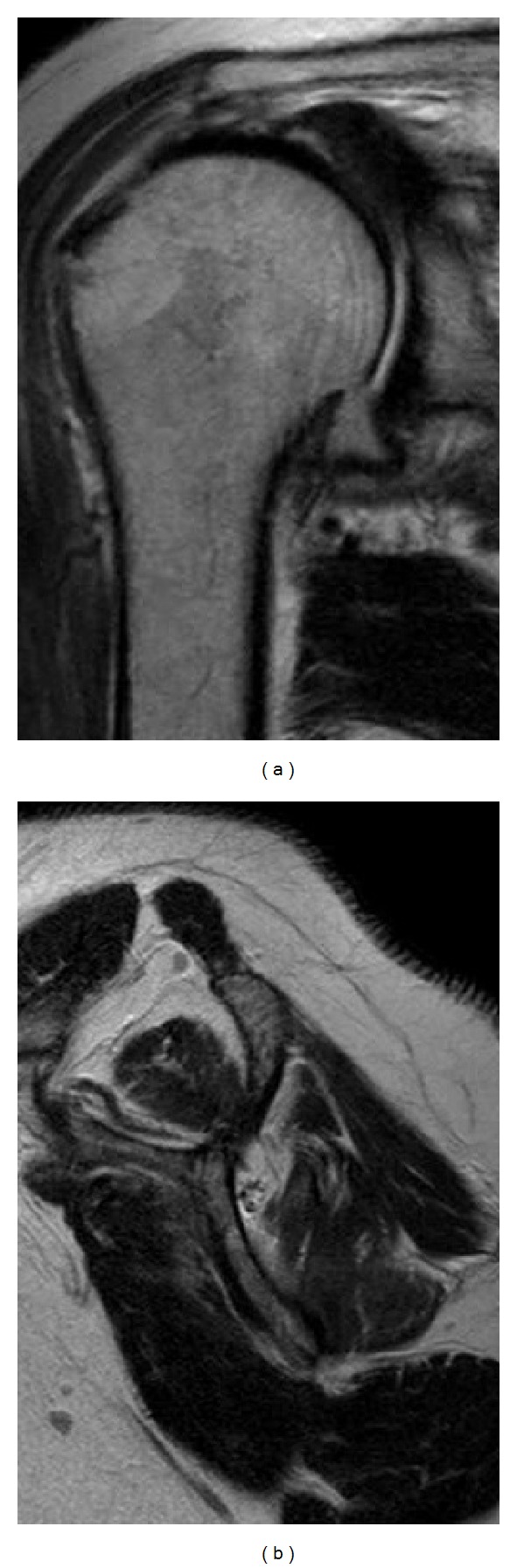
MRI imaging upon initial presentation to our hospital. (a) Coronal view of T2 shows a large tear approximately 3 cm long in the supraspinatus tendon. (b) Oblique sagittal view of T2 shows atrophy and fatty degeneration of the supraspinatus muscle.

**Figure 4:**
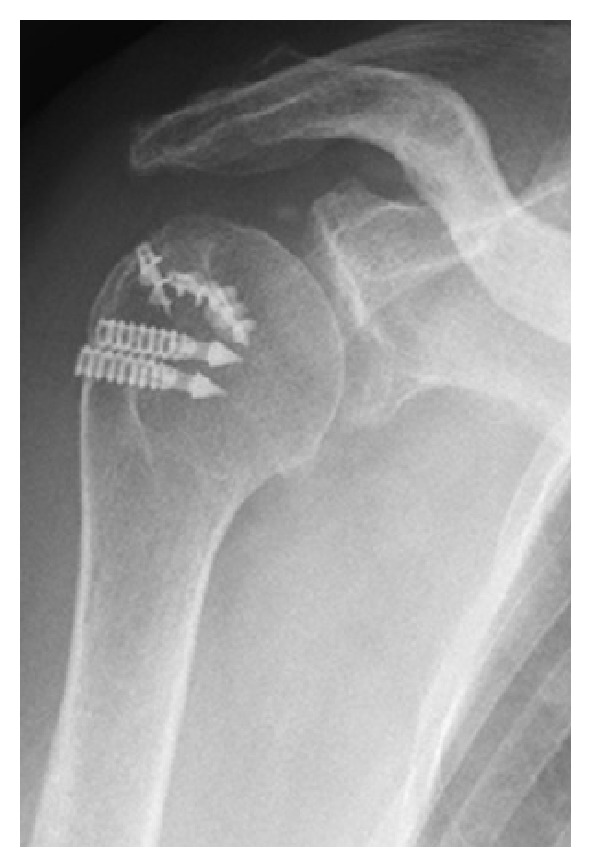
X-ray imaging after arthroscopic rotator cuff repair using a suture bridge technique.

A 61-year-old woman fell over a barrier curb while walking and bruised her right shoulder. A local clinic examined the patient and diagnosed her with anterior dislocation of the shoulder with a glenoid rim fracture (Figure 1(a)). The right shoulder had been immobilized with a sling for a month after immediate reduction (Figure 1(b)). However, the active range of motion for the shoulder remained limited. An MRI scan two months after injury revealed rupturing of the supraspinatus tendon. The patient was referred to our hospital for further management. Clinical examination found stiffness in the right shoulder with muscular weakness. Respective ranges for active and passive right shoulder flexion were 30 degrees and 120 degrees. There was sensory disturbance over the lateral aspect of the shoulder. Radiographs and CT scanning revealed a large anteroinferior glenoid rim fracture, a type IA fracture under the Ideberg classification [5, 6]. The articular surface was displaced by 2.5 mm, and the width of the bony fragment was 30% of the glenoid length. Upon initial presentation at our hospital, the fracture had already achieved partial union (Figure 2); therefore, we treated the glenoid rim fracture conservatively. An MRI scan showed a large tear approximately 3 cm long in the supraspinatus tendon, with atrophy and fatty degeneration of the supraspinatus muscle (Figure 3). Electromyography and nerve conduction studies showed an incomplete lesion of the axillary nerve; therefore, we diagnosed axillary nerve palsy in addition to glenoid rim fracture and rotator cuff tear. The axillary nerve palsy was incomplete and recovering. Following improvement in manual muscle testing (MMT) of the deltoid from grade 4/5 to 5/5 and the disappearance of the sensory disturbance three months after injury, we performed an arthroscopic rotator cuff repair using a suture bridge technique (Figure 4). Shoulder function recovered satisfactorily with approximately full ranges of motion one year after surgery. Constant score recovered from 38 points to 75 points.

## 3. Discussion

Despite anterior dislocations of the shoulder commonly associated with glenoid rim fractures, rotator cuff tears, and axillary nerve palsy, there is no reported case presenting all four injuries of the shoulder concomitantly. The incidence of axillary nerve palsy following anterior dislocation of the shoulder is 9-10% [7, 8]. The mechanism of axillary nerve injury in shoulder dislocations consists of traction and compression forces applied during stretching of the nerve across the humerus as it dislocates anteriorly. Prognosis for neurological recovery is reportedly excellent, as lesions associated with dislocations are typically either neuropraxic or axonotmetic lesions which are classified as class1 or class 2 of Sunderland classification [9, 10]. Therefore, early diagnosis and surgical repair of rotator cuff tears associated with shoulder dislocation is crucial, as early repairs favor improved outcomes compared with delayed repairs [3, 11]. Because of pain and swelling of the shoulder, it would be difficult to diagnose rotator cuff tears by physical remarks after dislocation of the shoulder. Since 14–63% of anterior dislocations are associated with rotator cuff tears [12], MRI should be performed when the patient has persistent pain or muscle weakness after reduction of the shoulder. Although Mall et al. reported that there was no indication that acute repair in traumatic injuries produced better outcomes [13], Simonich and Wright recommend proceeding with rotator cuff repair as soon as the diagnosis has been made in order to obtain the optimal results for terrible triad of injuries [14]. The tears of tendons of the rotator cuff cause atrophy and fatty degeneration of the rotator cuff muscles. These changes are irreversible and surgical repair is most effective for the prevention. The prognosis of unhappy triad depends essentially on brachial plexus recovery when the rotator cuff has been repaired early [15]. In this case, it is uncertain when the rotator cuff tear occurred. The prevalence of rotator cuff tear in the 60 s is 15.2% [16], and the incidence of rotator cuff tear with first-time anterior traumatic shoulder dislocation is 47.8% in the 60 s [17]. Although many elderly patients have symptomatic or asymptomatic rotator cuff tear, the prevalence rate of rotator cuff tear increases in association with dislocation of the shoulder. Moreover, she had not been suffering from shoulder symptoms before the injury. Therefore, the rotator cuff tear might be traumatic in this case.

A displacement threshold has been proposed for glenoid rim fractures, with a displacement of more than 4 mm of articular step-off, or more than 20% involvement of the glenoid, indicating that surgery is required [18]. In a cadaveric study, Itoi et al. reported that a defect with a width at least 21% of the glenoid length causes shoulder instability [19]. In the present case, the width of the bony fragment was 30% of the glenoid length; therefore, surgery may be indicated for the glenoid rim fracture according to Itoi et al. In contrast, Maquieira et al. reported that traumatic anterior shoulder dislocations with a large, displaced glenoid rim fracture can be successfully treated nonsurgically [6]. We did not perform osteosynthesis in the present case, as the fracture had already achieved union upon referral to our hospital.

There has been no reported case of the “terrible tetrad,” which consists of concurrent rotator cuff tear and axillary nerve palsy associated with anterior dislocation of the shoulder and a large glenoid rim fracture. This study presented a case of the “terrible tetrad" of the shoulder for the first time. We conclude that surgeons should consider an operation to repair the rotator cuff, in an attempt to prevent rotator cuff degeneration, if the symptoms of the shoulder such as pain or limited motion last after reduction of the dislocation and the nerve recovery is predicted.
